# Transplanting Fruit Trees and Evergreens

**Published:** 1855-03

**Authors:** 


### TRANSPLANTING FRUIT TREES AND EVERGREENS.

I consider the cultivation of fruits and flowers and evergreens as among the most agreeable, profitable, healthful, and refining of all occupations, and therefore transfer to the Journal some practical remarks of great value from an unknown source.

A tree should never be taken up whilst it will visibly shrink on removal. We have no criterion in the dropping of the leaves of the forest trees, as the origin of forest trees are so various, that many kinds require a certain degree of cold to stop their growth. Young nursery trees, too, being well cultivated, hold their leaves longer, and grow much longer than fruit bearing trees in orchards—so the proper time to transplant is whenever the juices of the tree become inactive. A dry summer, with an extreme degree of heat, followed by a delightful and seasonable autumn, prolonged into the heart of winter, has with us, added a third more wood of late growth of trees, and has, at the same time, delayed the season for transplanting.

In South Carolina, we find no difficulty in transplanting trees and shrubbery from November 25, to as late in the spring as we can retard the leaves. Trees should never be touched when the soil is frozen. The milder and dryer the weather in the winter, the better the success will be had. We dig our holes, after ploughing the land, as deep as we can; twelve inches deep, and at least five feet in diameter. We half fill these holes with good, rich compost, broken bones, &c., and then place the tree in its proper position, the earth in the hole being a little more elevated immediately under the trunk. We then place the roots so they are arranged in every part of the hole, when it is filled up carefully with a similar compost. The tree should not be planted more than one inch deeper than it stood in the nursery. When the hole is about three parts filled, we pour gently around the stem about five gallons of water, after which the operation is finished by completely filling it up, and making a slight mound around the trunk. We never pack in the earth around a tree, as the water will consolidate it sufficiently around the roots to make it grow. This watering will be all the tree will require, if it be properly mulched with leaves, straw, saw-dust, or old tan-bark. If trees have been long out of the ground, the roots should be well soaked six hours before planting, and we have frequently revived such as were to all appearance dead, by burying them entirely in the earth for ten days, after having restored vitality to the bark by soaking them in water. The trunks of newly transplanted trees should be protected from the sun. A bunch of broom sedge, so common everywhere in the South, if properly tied around, is the very best means of doing so. We head in all trees severely, no matter how fine the roots should be. Bearing trees should be prepared for removal one year previously, by cutting in both their heads and roots; but at best, the removal of large trees in the South is hazardous and unprofitable. Stakes to trees are useless. When a tree will not stand erect, it should be manured and cut in, until it requires sufficient vigor to stand alone. We should as soon tie a baby to a stake to make it stand, as a tree. The knife and food is all that is required to keep it erect and vigorous. Until newly planted trees are firmly rooted, they should be regularly inspected and straightened up. When watering is necessary, the earth should be removed for a few inches from the tree, and the water poured gently around the trunk, till the earth in the vicinity of the roots absorb it.

